# Supplementary figures and images for: Microbiota plasticity in tilapia gut revealed by meta-analysis evaluating the effect of probiotics, prebiotics, and biofloc
Source: PeerJ. 2023 Oct 11;11:e16213. doi: 10.7717/peerj.16213 (PMC10576497; doi:10.7717/peerj.16213)

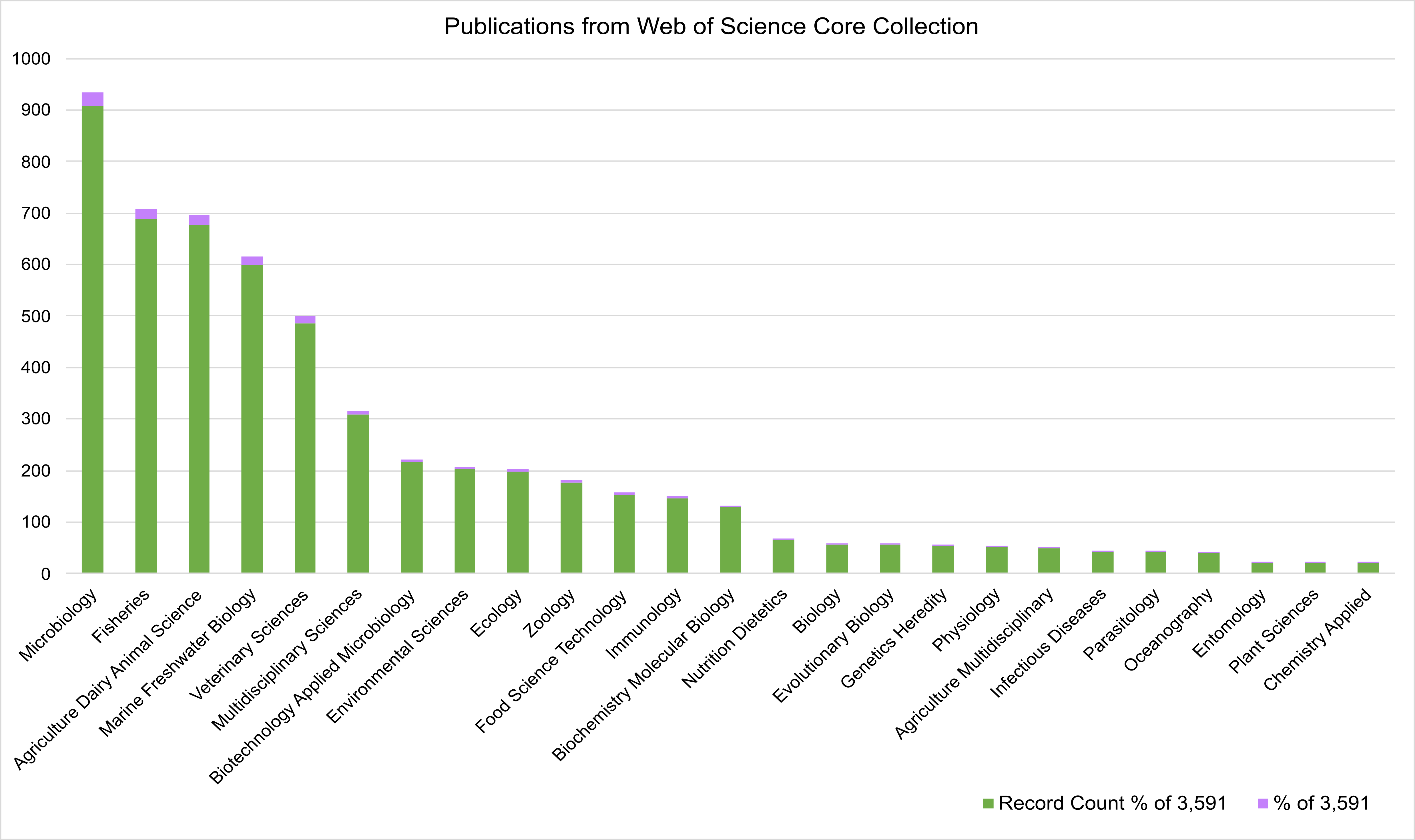

Supplement: Supplemental Information 1 [file peerj-11-16213-s001.png]

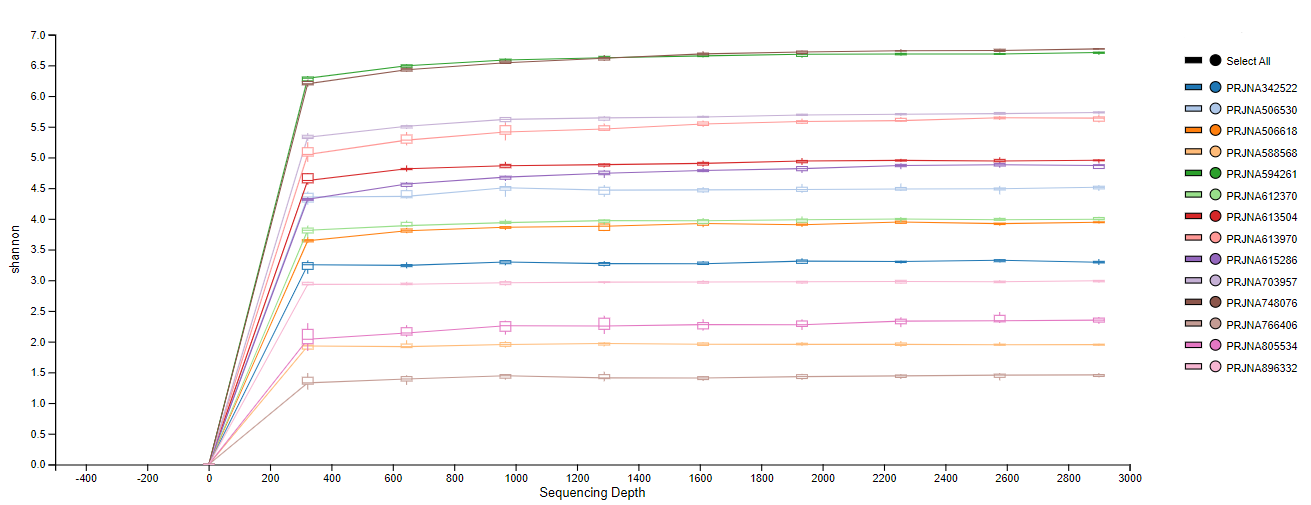

Supplement: Supplemental Information 2 [file peerj-11-16213-s002.png]

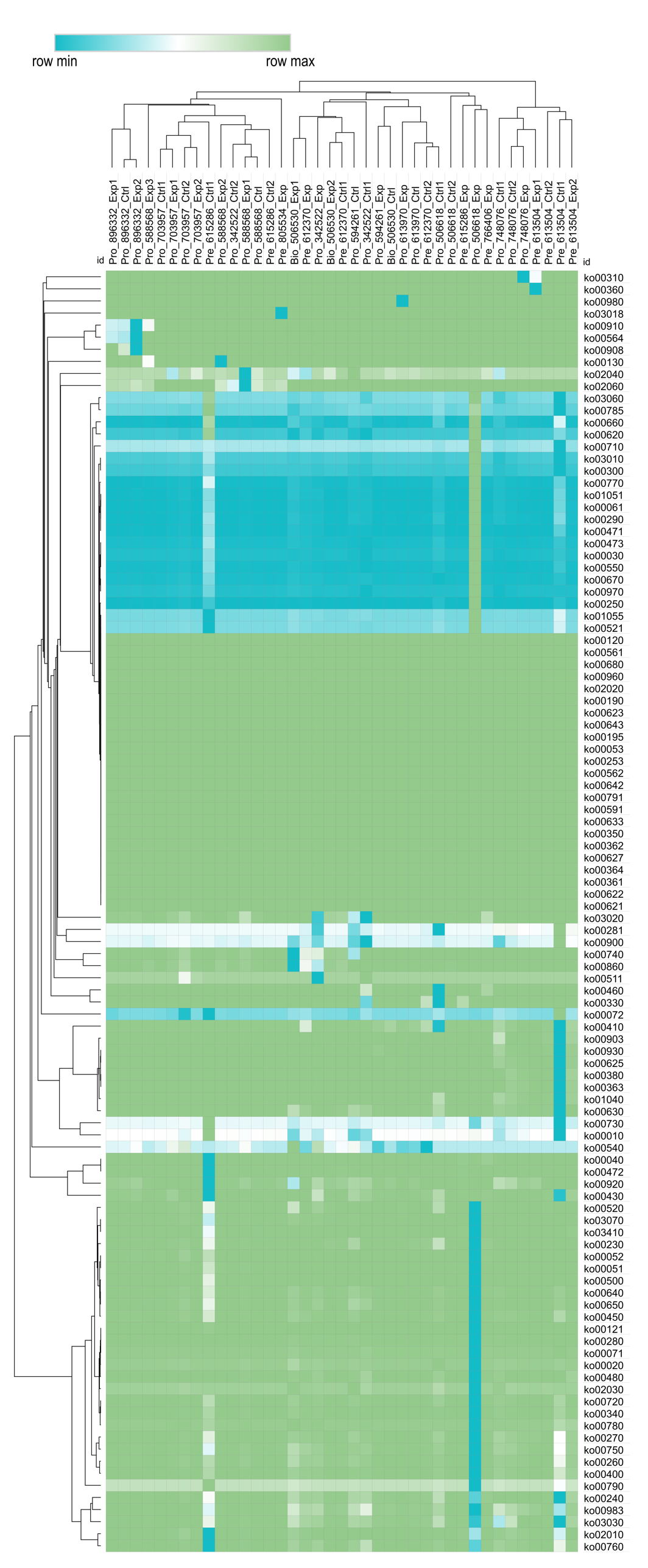

Supplement: Supplemental Information 3 [file peerj-11-16213-s003.png]

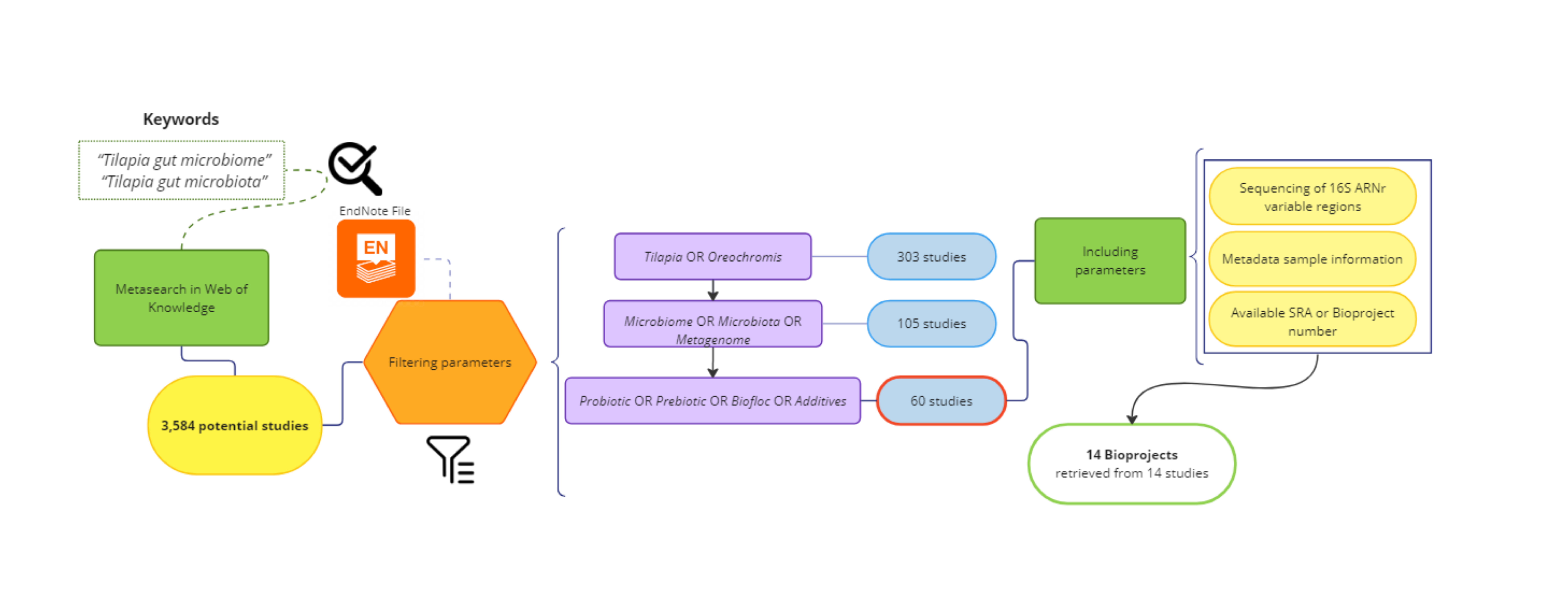

Supplement: Supplemental Information 4 [file peerj-11-16213-s004.png]
